# Evaluation of Tricaine (MS-222) and Eugenol for Sedation of Peruvian Grunt *Anisotremus scapularis*

**DOI:** 10.3390/ani15091322

**Published:** 2025-05-02

**Authors:** Luis Antonio Espinoza-Ramos, Ydelsa Puma-Vilca, Jordan I. Huanacuni, Renzo Pepe-Victoriano

**Affiliations:** 1 Escuela de Ingeniería Pesquera, Universidad Nacional Jorge Basadre Grohmann, Tacna 23000, Peru; ydelsapumavilca@gmail.com; 2 Área de Biología Marina y Acuicultura, Facultad de Recursos Naturales Renovables, Universidad Arturo Prat, Iquique 101000, Chile; jordan.92ihp@gmail.com; 3 Programa de Magíster en Acuicultura, Mención en Cultivos de Recursos Hidrobiológicos y Mención en Acuaponia, Facultad de Recursos Naturales Renovables, Universidad Arturo Prat, Arica 1031597, Chile; 4 Finfish Aquaculture Sociedad Anónima Cerrada, Tacna 23004, Peru; 5 Núcleo de Investigaciȯn Aplicada e Innovaciȯn en Ciencias Biolȯgicas, Facultad de Recursos Naturales Renovables, Universidad Arturo Prat, Arica 1031597, Chile

**Keywords:** anesthetics, sargo, Chita, induction, recovery, clove oil, methanesulfonate

## Abstract

*Anisotremus scapularis* is a commercially important species in Peru, where its cultivation and management require technological strategies to minimize stress during aquaculture and fishing practices. Fish handling and transport can induce adverse physiological responses, making anesthetic use a key tool to mitigate these effects and enhance animal welfare. However, information on optimal doses, safety margins, and induction and recovery times of anesthetics in this species remains limited. This study evaluated the effects of eugenol and tricaine (MS-222) on the sedation of *A. scapularis juveniles*. A total of 450 individuals (9–10 cm total length) were exposed to different concentrations of eugenol and MS-222 (20, 40, 60, 80, and 100 mg/L) via immersion. Induction and recovery times were recorded, determining that the lowest concentrations allowing an optimal induction time (<6 min) and adequate recovery (<16 min) were 20–60 mg/L for eugenol and 80 mg/L for MS-222. These results provide essential information to optimize sedation protocols for *A. scapularis juveniles*, promoting more efficient handling with minimal impact on fish welfare in production and fishery management systems.

## Simple Summary

This study evaluated the anesthetic effect of eugenol and tricaine methanesulfonate (MS-222) on juvenile Peruvian grunt (*Anisotremus scapularis*), an important species for aquaculture in the southeastern Pacific. The objective was to compare induction and recovery times at different concentrations of both anesthetics. Fish were exposed to varying doses of eugenol (25, 50, 75, 100 mg/L) and MS-222 (50, 100, 150, 200 mg/L). Eugenol induced anesthesia faster and at lower concentrations than MS-222; however, recovery times were longer. These results suggest that while eugenol is effective for rapid induction, MS-222 may be preferable when faster recovery is needed. Further research is needed to evaluate the physiological effects and safety of these anesthetics in this species.

## 1. Introduction

Aquaculture in Peru has experienced an annual growth rate of 20% in recent years, becoming a prominent activity due to its high production levels [1]. On the Peruvian coast, *Anisotremus scapularis* is a commercially valuable species because of its culinary qualities, and efforts are currently underway to achieve conditioning and captive breeding [2,3,4]. *A. scapularis*, commonly known as Peruvian grunt, belongs to the Haemulidae family, characterized by coastal marine species that emit characteristic sounds by contracting muscles associated with the swim bladder [5]. This species is distributed in the southeastern Pacific, mainly from northern Peru to northern Chile, and inhabits rocky areas and sandy bottoms, commonly between 5 and 50 m deep [6]. It reaches sexual maturity between 2 and 3 years of age, with an average size at sexual maturity of close to 20 cm in total length [7]. Its reproduction is seasonal, occurring mainly in spring and summer, when there are increases in temperature and productivity in the coastal zone [5]. Adults can reach sizes of up to 40 cm, with the commercial harvest size generally being between 25 and 30 cm [5]. It feeds on small benthic invertebrates, such as crustaceans, molluscs and polychaetes. A long-distance migratory pattern has not been described, although some seasonal displacement associated with environmental conditions has been observed [7]. Although successful reproduction has already been achieved under controlled conditions [8], further basic research is needed to optimize its management across different farming stages.

Among the key tools for proper management are anesthetics, which help reduce stress during common practices such as blood sampling, handling, vaccination, treatments, transportation, and induced spawning [9]. Stress in fish is often triggered during handling and transport [10,11], and can also be caused by physicochemical factors such as salinity, temperature, pH, and dissolved oxygen [12,13,14,15], negatively affecting their immune system and increasing mortality rates [16].

The use of anesthetics in aquaculture is regulated and must meet safety and efficacy standards [17]. These substances can be synthetic or plant-derived [16], with commonly used options including MS-222, benzocaine, quinaldine, 2-phenoxyethanol, and clove oil (whose active compound is eugenol) [15]. In aquaculture, anesthetics are used frequently because they considerably facilitate the various procedures mentioned earlier. These procedures have the potential to affect the commercial production of fish [18,19,20,21,22,23]. However, many of these compounds present limitations related to cost, availability, and commercial restrictions [24]. The limited availability of approved anesthetics has led to the frequent use of unauthorized products, posing risks to both fish and human health.

In Peru, there is currently no specific national regulation that governs the use of anesthetics in aquaculture. However, general guidelines for veterinary pharmaceutical products in aquaculture fall under the supervision of the Servicio Nacional de Sanidad Agraria del Perú (SENASA). In the absence of specific legislation, substances such as eugenol are used in experimental and production settings following international references and best practices, particularly those recommended by entities such as the Food and Agriculture Organization (FAO) and OIE (World Organisation for Animal Health). For approval, anesthetics must undergo rigorous evaluations, considering their effectiveness, toxicity, and adverse effects, including potential genotoxicity [25]. Their performance depends on biological (species, size, reproductive stage) and environmental (temperature, salinity, pH) variables, which influence induction and recovery times [26,27].

*A. scapularis*, commonly known as Peruvian grunt, belongs to the family Haemulidae, characterized by coastal marine species that emit characteristic sounds through the contraction of muscles associated with the swim bladder [5]. This species is distributed in the southeastern Pacific, mainly from northern Peru to northern Chile, and inhabits rocky areas and sandy bottoms, commonly between 5 and 50 m deep [6]. It reaches sexual maturity between 2 and 3 years of age, with an average size at sexual maturity close to 20 cm in total length [7]. Reproduction is seasonal, occurring mainly in spring and summer, when there are increases in temperature and productivity in the coastal zone [8].

This study evaluated the effects of eugenol and tricaine (MS-222) on juveniles of *A. scapularis*, focusing on procedures where anesthetic use can prevent physical damage and minimize stress and its physiological consequences. The appropriate use of anesthetics is essential for handling, transportation, and experimental procedures in fish, with stress control being a critical factor in aquaculture research [28].

## 2. Materials and Methods

The methodology used in this study has been previously validated in other works [24,29,30], where it demonstrated its effectiveness in the evaluation and management of the studied variables. Based on this background, the methodology was refined and adapted to meet our specific needs and the context in which the research was conducted, ensuring its applicability under the conditions of the study.

### 2.1. Conditioning of Juvenile A. scapularis

This study was conducted at the Morro Sama Aquaculture Center (CAMOSA), located 75 km from the Tacna–Ilo Coastal Highway, in the town of Morro Sama in the coastal district of Sama Las Yaras, in the Tacna region (18°00′08″ S–70°53′21″ W). The juvenile *A. scapularis* underwent a 24 h fasting period prior to biometric measurements, and individuals with a length between 9 and 10 cm were selected (length: 9.44 ± 0.37 cm, weight: 17.13 ± 2.02 g). They were then transferred to the laboratory, where they remained in a 1 m diameter, 800 L fiberglass circular tank, undergoing a two-week acclimatization period. During this acclimatization period, the water parameters were maintained within a specific range: pH between 7 and 8, temperature of 17 to 18 °C, and oxygen concentration of 7 to 8 mg/L. These conditions were established with the aim of minimizing stress, replicating normal conditions, and ensuring the validity of the experiment.

Seawater was used for the conditioning process. It was passed through a filtration system with 20, 10, and 5 μm filters. Additionally, the water used for induction and recovery underwent the same filtration process, followed by ultraviolet (UV) radiation treatment. The salinity of the seawater used throughout the experiment was approximately 35‰.

During the acclimatization period, the animals were fed handmade food three times a day. This food consisted of 50% protein. As part of the preparation for the experimental exposures, the fish were subjected to a 24 h fasting period.

In the experiments with eugenol (Prevest, 70%) and tricaine (Syndel, 100%), five different anesthetic doses (20, 40, 60, 80, and 100 mg/L) were used, each with three replicates, employing 6 L tanks with 15 individuals per tank (43 kg/m^3^). Eugenol was diluted with 90% ethanol in a 1:10 ratio, and this mixture was poured into the designated ‘anesthetic tanks’. The entire process was conducted under controlled conditions.

All animals were exposed to the anesthetic prior to water renewal, ensuring consistent exposure conditions across all replicates. The renewal process was carried out immediately after this initial exposure to maintain water quality and anesthetic concentration stability throughout the trial.

### 2.2. Determination of Anesthetic Induction Time

To determine the anesthetic induction time, different anesthetic stages were evaluated in the fish following established parameters based on those described by Ross & Ross [24] (Table 1).

The induction time was defined as the period from the moment the fish encountered the anesthetic solution until it exhibited a loss of body movement. For this, the fish were transferred to the induction tank, which contained the water and anesthetic mixture. From this point onward, the changes in the behavior of the fish under study were closely observed, evaluating parameters such as swimming axis, body mobility, opercular frequency and rhythm, as well as responses to external mechanical stimuli and reflex responses. Anesthesia exposure durations were accurately recorded using a stopwatch.

### 2.3. Determination of Anesthetic Recovery Time

To determine the recovery time, the modified scale of Ross and Ross [24] (Table 1) was followed. This time was established as the interval from the removal of the fish from the anesthetic solution during the induction phase (I-3) to its placement in the recovery tank, considering the various stages of recovery. The time required for the fish to fully recover from the anesthetic effect was recorded.

### 2.4. Data Analysis

Statistical analyses were performed using Rstudio statistical software version 2024.04.2+764 from Rstudio, Inc (Boston, MA, USA). All data were subjected to normality tests using the Anderson–Darling test and variance homogeneity using Bartlett’s test. Data were evaluated with one-way analysis of variance (ANOVA) and Tukey’s post hoc test. When normality assumptions were not met, data were analyzed using the Kruskal–Wallis test to assess differences between treatments and the Dunnett test to determine homogeneous groups. The Pearson or Spearman correlation coefficient was also applied as appropriate. The experimental graphs were created using the ggplot2 package.

## 3. Results

During the experiment and two weeks post-anesthesia, no mortalities were recorded in *A. scapularis*.

### 3.1. Eugenol

Stage I-1 and R-1 showed a normal distribution (A = 0.599, *p* = 0.098 and A = 0.434, *p* = 0.262, respectively). The induction times during the anesthetic sedation stages in juvenile *A. scapularis* exhibited a dose-dependent variation with eugenol anesthetic, indicated by the correlation coefficient (Table 2), meaning that the induction time decreased with increasing eugenol concentration at each anesthetic stage. Furthermore, the animals exposed to this anesthetic reached accelerated anesthesia at 80 mg/L (96.00 s ± 14.41). However, at concentrations of 40 mg/L and 60 mg/L, no significant differences were observed among the three evaluation stages (I-1, I-2, and I-3).

Regarding recovery time, it can be observed that at concentrations of 40 mg/L and 60 mg/L, there was no significant difference during stages R-1 and R-2. However, at concentrations of 80 mg/L and 100 mg/L, there was a significant difference in each stage (R-1, R-2, and R-3).

**Figure 1:**
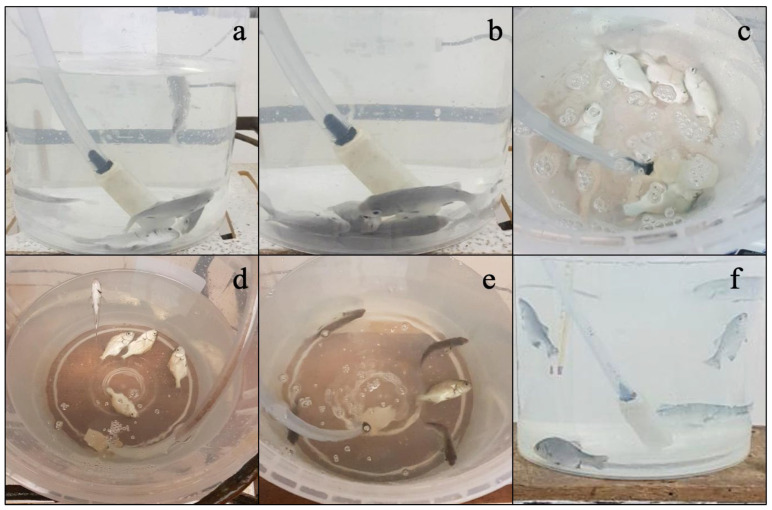
Anesthetic induction response. (**a**) Stage I, (**b**) Stage II, and (**c**) Stage III and anesthetic recovery (**d**) Stage I, (**e**) Stage II, and (**f**) Stage III in *A. scapularis*.

Figure 1 shows that the fish subjected to the anesthetic induction process experience an alteration in their swimming ability, manifested by the loss of flotation axis, irregular movements, and a marked increase in opercular frequency (Figure 1a). In a more advanced phase, a progressive reduction in body mobility is observed along with a decrease in opercular activity, which remains in a stable pattern, as well as the absence of a response to external stimuli (Figure 1b). Finally, in the deepest stage of anesthesia, the fish present total immobility, accompanied by severe depression of opercular activity and an irregular opercular rhythm (Figure 1c).

During recovery, the fish maintained a state of immobility with a progressive increase in opercular frequency and lack of response to stimuli (Figure 1d). Recovery was defined as the reactivation of body movement and response to stimuli (Figure 1e). In the final stage of recovery (R-3), the fish took longer to recover their usual swimming behavior (Figure 1f).

**Figure 2:**
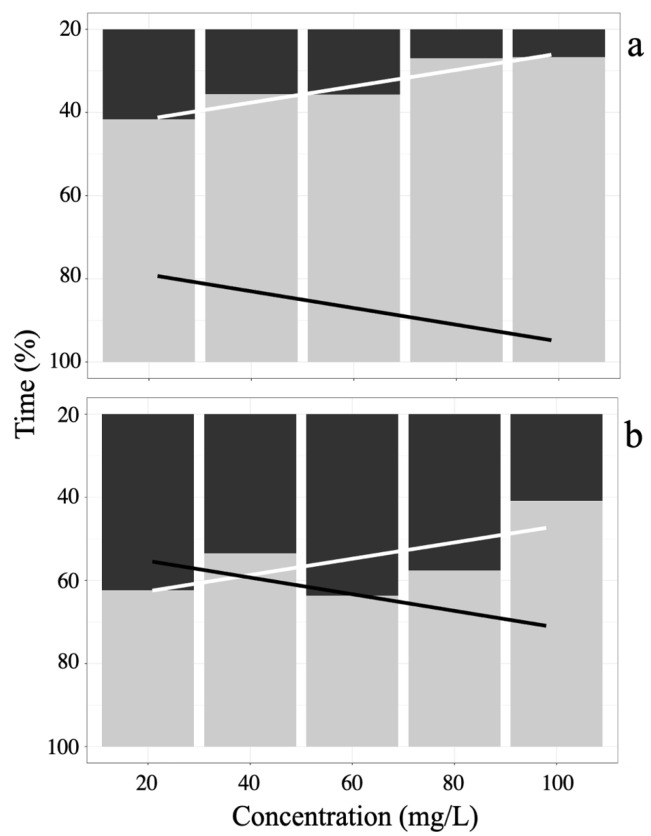
Percentage anesthesia time in *A. scapularis* with (**a**) Eugenol, and (**b**) Tricaine MS-222. Induction state (black bars) and its trend line (black line), anesthetic recovery state (gray bars) and its trend line (white line).

Induction times during sedation and anesthesia stages in juvenile *A. scapularis* showed a dose-dependent effect of eugenol concentration, as shown in Figure 2a, where the 100 mg/L concentration presents the shortest induction time (8.3%, 79.67 s ± 6.66), while the induction time at 20 mg/L (27.1%, 237.67 s ± 43.36) shows the longest induction time, with a negative trend. Additionally, it is observed that at the 100 mg/L concentration, the recovery time (91.7%, 881.33 s ± 6.11) is higher than the recovery time at the 40 mg/L concentration (72.9%, 858.67 ± 23.35) with a positive trend.

### 3.2. Tricaine MS-222

The R-1 and R-3 stages followed a normal distribution (A = 0.413, *p* = 0.294 and A = 0.585, *p* = 0.107, respectively). The induction times in juvenile *A. scapularis* showed a dose-dependent variation of the anesthetic MS-222, indicated by the correlation coefficient (Table 3), with the concentrations of 20 mg/L and 40 mg/L showing no effect during the I-2 and I-3 stages, indicating no significant difference between these two stages. The 60 mg/L concentration showed the longest induction time (352.33 s ± 30.52), with a prolonged I-3 stage (204.00 ± 9.64). Recovery time in the R-1 stage showed no significant difference among treatments (F = 0.5362, *p* = 0.477).

As shown in Figure 1, the anesthetic induction process in fish generates a series of behavioral responses, initially characterized by the loss of postural control, erratic movements, and an increase in opercular frequency (Figure 1a). As the anesthetic effect progresses, there is a decrease in body mobility, along with a reduction in opercular frequency that maintains a stable pattern and a lack of response to stimuli (Figure 1b). In the I-3 stage, commonly referred to as the deep anesthesia phase, the fish reach complete immobility, accompanied by a marked reduction in opercular activity and altered respiratory rhythm (Figure 1c).

In the recovery phase (R-1), no differences were observed in the recovery patterns across all treatments, characterized by persistent immobility, a progressive increase in opercular frequency, and a lack of response to stimuli (Figure 1d). In the R-2 stage, late reactivation of mobility and recovery of response to mechanical stimuli were observed (Figure 1e). Finally, in the R-3 stage, the fish took longer to restore swimming axis control and to reach behavior like that observed before anesthesia (Figure 1f).

## 4. Discussion

The exposure of juvenile *A. scapularis* to the anesthetics eugenol and tricaine (MS-222) demonstrated a differential response in anesthetic induction and recovery. The evaluation of anesthetic stages was conducted following standardized methodologies described by McFarland [31], Hikasa et al. [32], Iwama [33], Cooke et al. [34], and Ross and Ross [24] with the anesthetic stage characteristics in *A. scapularis* being consistent with those previously reported by these and other authors (Table 4).

### 4.1. Eugenol

Studies by Gomes et al. [46], Meinertz et al. [47], and Cupp et al. [48,49] highlight that eugenol, the main active component of clove oil (70–90% by weight), has been established as an effective anesthetic for numerous species of teleost fish [50]. The induction times for the sedation and anesthesia stages in juvenile *A. scapularis* showed a dose-dependent variation with eugenol (Figure 2).

These results suggest that higher concentrations of the anesthetic result in shorter induction times, a claim supported by previous research [51], which indicates that concentration is inversely proportional to induction time.

Moreover, the recovery time was greater than the anesthetic induction time, confirming that higher concentrations lead to a longer recovery time in fish. These findings are consistent with previous studies that highlight the relationship between eugenol concentration and recovery duration [50].

Other studies, such as those by He et al. [9], provided information on induction and recovery times for adult spotted sea bass (*Lateolabrax maculatus*), indicating that effective concentrations for eugenol were 60 mg/L. Similar results were obtained for *A. scapularis* in this study, with longer times for deep sedation (I-3) at concentrations of 20, 40, and 60 mg/L eugenol.

Additionally, Woody et al. [52] demonstrated that eugenol is useful as an anesthetic in red salmon (*Oncorhynchus nerka*), achieving anesthesia induction with 50 mg/L and surgical anesthesia with 80 mg/L in less than three minutes, with no mortality cases. Moreover, Cunha and Rosa [50] found that concentrations of 20 to 60 mg/L eugenol were effective for immobilizing seven species of tropical reef fish, with short induction times (<2 min) and recovery times of 2 to 4 min, directly related to anesthetic concentration. In our work, *A. scapularis* showed anesthetic induction times (<4 min) and recovery times ranging from 11 to 17 min.

He et al. [9] evaluated the acute toxicity of eugenol and benzocaine and the estimated induction and recovery times. Additionally, the anesthetic efficacy of different concentrations of eugenol at water temperatures of 20 and 30 °C was compared. They concluded that eugenol was an effective anesthetic, but its addition did not improve the transport of sea bass. Purbosari et al. [53] explored both natural and synthetic anesthetics derived from terrestrial and aquatic resources. This review of the different types, sources, and applications of natural and synthetic anesthetics was used to investigate the potential of marine algae as an anesthetic and the advantages and disadvantages of anesthetics. Uehara et al. [54] and Weber et al. [55] evaluated the efficacy of four anesthetic agents (including eugenol and MS-222) in *Oligosarcus argenteus* and *Solea senegalensis*. The conclusion of these two studies was that all the anesthetics tested effectively induced anesthesia in both fish species. Jerez-Cepa et al. [56] evaluated the effects of sedation doses of two anesthetics, clove oil (CO) and MS-222, on markers related to the regulation of the HPI (hypothalamic–pituitary–interrenal) axis and energy management in juvenile gilthead seabream (*Sparus aurata*) following simulated transport and subsequent recovery. Both anesthetics induced changes in the energy resource management of *S. aurata*.

Thus, eugenol presents itself as an alternative for the sedation and anesthesia of fish due to its low associated toxicity. However, it is important to determine the appropriate concentrations for the specific purpose of the experiment, as studies have reported toxicity and subsequent mortality in other teleosts when inappropriate concentrations were used [57]. Although eugenol proved effective as an anesthetic agent at low concentrations, several studies have reported potential toxic effects at higher concentrations or prolonged exposures, including damage to gill tissues, oxidative stress, and genotoxicity in teleost fish [25,49]. In addition, its use in fish intended for human consumption is not yet approved by regulatory agencies such as the FDA, which limits its commercial application in many countries. Therefore, it is essential to determine safe sublethal levels for this species through additional studies addressing both acute and chronic toxicity.

Although MS-222 is FDA approved for use in fish intended for human consumption, it requires a mandatory 21-day waiting period before slaughter [58,59], which may limit its applicability in certain production systems where rapid processing is needed.

### 4.2. MS-222 (Tricaine)

The results of this study support the assertion that higher concentrations of the anesthetic lead to shorter induction times, which is consistent with previous research [51] that suggest that the anesthetic concentration is inversely proportional to induction time.

In line with these findings, Uehara et al. [54] conducted studies demonstrating the efficacy of various anesthetics in inducing anesthesia in lambari-bocarra (*Oligosarcus argenteus*), highlighting that concentrations of 75 and 100 mg/L of MS-222 induced deep anesthesia. In our study, 60 and 80 mg/L induced a longer period of deep anesthesia (I-3).

On the other hand, ref. [60] simulated the transport of *A. scapularis* using tricaine (MS-222) for 24 h and recommended transporting them at 19 °C with MS-222 at 15 mg/L. In contrast, in our study, *A. scapularis* showed the longest deep sedation time at 60 mg/L. This difference in suggested concentrations is likely due to the exposure time to the anesthetic, as our experiment aimed for deep anesthesia, unlike the goal of Rosado et al. [60], who aimed for low sedation that would allow for transport and high survival of the species. However, other studies have established that the effective dose of MS-222 for fish is 100 mg/L [61,62,63,64,65,66]. Additionally, some fish species require higher concentrations (140–150 mg/L) of MS-222 to achieve the desired induction (≤3 min) and recovery (≤5 min) times [9,67,68], which are similar to those recorded in our study, with induction times 2.5–6 min and recovery times 4.3–7 min.

Of the two anesthetics studied, eugenol has a greater variability in effective concentrations according to the species of fish. Although MS-222 is FDA approved for use in fish intended for human consumption, it requires a mandatory 21-day waiting period before slaughter [58,59], which may limit its applicability in certain production systems where rapid processing is needed.

In addition, MS-222 has been considered more dangerous, with reports of potential ocular and neurological disorders in fish, amphibians, and humans with chronic exposure, although no recent incidents have been reported [69]. However, chemical anesthesia remains the only approved method for anesthetizing fish during sampling and surgical procedures according to various organizations [70]. Although MS-222 is one of the most used and approved anesthetics in aquaculture, its toxicity has also been widely documented, especially at high concentrations or following prolonged exposure. Adverse effects such as mucosal irritation, neurological alterations, and respiratory depression have been reported in several species [64,69]. Furthermore, its use requires a mandatory withdrawal period before the fish can be consumed, which adds complexity to its practical application. These aspects must be considered when selecting the most appropriate anesthetic according to the specific purpose of the aquaculture procedure.

### 4.3. Perspectives and Development of Anesthetics in Aquaculture

The use of anesthetics in modern aquaculture remains an area of ongoing research, driven by the need to optimize animal welfare. In the case of *Anisotremus scapularis*, anesthesia could be especially beneficial during routine aquaculture procedures, such as biometric measurements, vaccination, selection, transport, and tissue sampling. These activities, when performed without sedation, can trigger acute stress responses that affect survival, growth, and immune function. Applying effective anesthetic protocols during these procedures can significantly improve fish welfare and the overall success of aquaculture operations [71]. Traditional anesthetics such as eugenol and MS-222 have proven effective in various species, including *Anisotremus scapularis*. However, they have limitations in terms of regulation, toxicity, and variability in response depending on the species and environmental conditions.

One of the main lines of development in this field is the search for alternative anesthetics that are safer, more effective, and environmentally sustainable. Recent studies have explored the use of synthetic anesthetics and natural compounds derived from terrestrial and aquatic resources [53], which show potential as sedatives in fish due to their hypnotic properties and lower environmental impact. Some algae species, such as *Ecklonia cava* [72], *Eucheuma cottonii* [53], and *Inyegaria estrellada* [73], have been identified as sources of bioactive compounds with anesthetic effects. However, more research is needed to evaluate their efficacy, optimal dosages, and potential adverse effects in aquaculture species of interest.

On the other hand, the development of synthetic anesthetics with improved safety profiles is also a promising line of research. New compounds could be designed to minimize side effects and reduce post-anesthesia recovery time, thus improving the efficiency of fish handling. Furthermore, innovative approaches such as the use of nanoencapsulation to prolong the anesthetics’ effects and control their release have been proposed, enabling more precise and safe application in intensive production systems [74].

Finally, the future of anesthesia in aquaculture not only depends on the development of new compounds but also on optimizing their application. Combining anesthetics with management strategies, such as water temperature control or supplementation with anti-stress additives in feed, could enhance their effectiveness and reduce reliance on potentially harmful chemicals. In this regard, future studies will focus on identifying more effective, safer, and economical natural anesthetics for fish species of economic importance, both ornamental and for consumption, encompassing different sizes and ages [53].

Therefore, the development of anesthetics in aquaculture should be directed toward safer, more efficient, and sustainable alternatives. Research into natural compounds, the improvement of synthetic formulations, and the optimization of application protocols will be key to ensuring the welfare of fish and the economic and environmental viability of the aquaculture industry. Despite the results obtained in this study, we acknowledge that physiological health parameters of the fish following exposure to the anesthetics were not evaluated. Therefore, future studies will include the analysis of stress indicators such as cortisol and glucose, as well as hematological and genotoxic parameters, in order to establish the safety of these substances for sustained use in aquaculture. In addition, future research should evaluate the actual potential of anesthetics to modulate the hypothalamic–pituitary–interrenal (HPI) axis and reduce cortisol release, which plays a critical role in the fish stress response [75]. Understanding these physiological interactions will provide better guidance for selecting anesthetics that not only reduce behavioral indicators of stress, but also minimize the physiological impact on fish health.

## 5. Conclusions

- We determined that an effective concentration of eugenol in the range of 20 to 60 mg/L and 80 mg/L of MS-222 was sufficient to achieve induction and anesthetic recovery times of less than 6 and 16 min, respectively, with no observed mortality.
- In this study, eugenol was a more effective anesthetic than MS-222. This efficacy was defined as a combination of: (a) short induction times, (b) complete recovery with no visible adverse effects, and (c) an adequate duration of deep anesthesia (stage I-3) that allowed handling procedures to be performed with minimal interference from fish movement. In addition, eugenol showed better performance in terms of stability and duration of the deep anesthetic state.

## Figures and Tables

**Table 1:** Response to anesthetic induction and recovery at different stages in *A. scapaularis*. Adapted from Ross and Ross [24].

Anesthesia	Stage Code	Description
Induction	I-1	Loss of swimming axis
		Erratic swimming
		Accelerated opercular frequency
	I-2	Loss of body movement
		Decreased opercular frequency
		Regular opercular rhythm
		Absence of response to external mechanical stimulus
		Absence of reflex response
	I-3	Body immobility
		Severe decrease in opercular frequency
		Irregular opercular rhythm
Recovery	R-1	Body immobility
		Increased opercular frequency
		Regular opercular rhythm
		Absence of response to external mechanical stimulus
	R-2	Onset of body movement
		Response to external mechanical stimulus
	R-3	Recovery of swimming axis
		Behavior like that of pre-anesthesia

**Table 2:** Anesthetic induction and recovery times (s, mean ± SD), and correlation coefficient (r) in juvenile *A. scapularis* at different concentrations of eugenol. Different letters indicate significant differences (one-way ANOVA, *p* < 0.05).

	Eugenol Concentrations (mg/L)					Correlation	
Stage	20	40	60	80	100	*p*-Value	*r*
I-1	52.00 ± 3.61 a	42.67 ± 5.03 b	40.33 ± 5.13 b	20.33 ± 3.51 c	15.67 ± 2.08 c	<0.001	−0.942
I-2	82.33 ± 19.40 a	71.33 ± 6.66 ab	86.33 ± 10.69 a	31.33 ± 2.52 b	29.00 ± 4.36 b	0.003	−0.715
I-3	103.33 ± 20.43 a	94.33 ± 20.60 ab	100.67 ± 9.29 ab	44.33 ± 8.39 bc	35.00 ± 3.61 c	<0.001	−0.840
I-Total	237.67 ± 43.36 a	208.33 ± 18.77 ab	227.33 ± 23.12 a	96 ± 3.46 bc	79.67 ± 6.66 c	<0.001	−0.859
R-1	79.33 ± 17.01 c	104.00 ± 10.58 bc	101.33 ± 4.16 bc	147.00 ± 4.00 a	114.67 ± 2.08 b	0.005	0.688
R-2	170.00 ± 13.23 c	229.00 ± 9.00 ab	232.67 ± 4.62 ab	258.00 ± 19.08 a	218.33 ± 10.60 bc	0.159	0.383
R-3	390.67 ± 9.02 c	525.67 ± 4.04 bc	600.00 ± 9.17 a	597.67 ± 2.52 a	548.33 ± 9.07 b	0.010	0.639
R-Total	640 ± 38.69 d	858.67 ± 23.35 c	934.00 ± 12.17 b	1002.67 ± 15.70 a	881.33 ± 6.11 c	0.187	−0.360

**Table 3:** Induction and recovery anesthesia times (s, mean ± SD), and correlation coefficient (r) in juvenile *A. scapularis* at different concentrations of MS-222. Different letters indicate significant differences (one-way ANOVA, *p* < 0.05).

	MS-222 Concentrations (mg/L)					Correlation	
Stage	20	40	60	80	100	*p*-Value	*r*
I-1	300.33 ± 13.65 a	186.67 ± 9.29 b	50.33 ± 3.79 c	41.67 ± 3.79 d	24.33 ± 3.21 e	<0.001	−0.977
I-2	0.00 ± 0.00 c	0.00 ± 0.00 c	98.00 ± 17.09 a	110.67 ± 7.37 a	53.00 ± 1.00 b	0.011	0.637
I-3	0.00 ± 0.00 d	0.00 ± 0.00 d	204.00 ± 9.64 a	139.33 ± 11.37 b	72.67 ± 3.06 c	0.031	0.558
I-Total	300.33 ± 13.65 ab	186.67 ± 9.29 bc	352.33 ± 25.42 a	291.67 ± 21.96 ab	150.00 ± 6.56 c	0.003	0.718
R-1	21.00 ± 2.65 a	19.33 ± 5.13 a	21.33 ± 17.93 a	19.33 ± 4.04 a	26.33 ± 3.21 a	0.477	0.199
R-2	53.33 ± 1.53 bc	51.33 ± 3.21 c	51.33 ± 5.69 b c	74.67 ± 5.03 ab	114.67 ± 0.08 a	0.001	0.771
R-3	192.00 ± 3.00 c	188.33 ± 7.64 c	220.67 ± 15.50 b	234.33 ± 5.13 ab	284.33 ± 13.65 a	<0.001	0.912
R-Total	266.33 ± 5.69 bc	259.00 ± 15.10 c	293.33 ± 30.24 ab	328.33 ± 14.05 ab	425 ± 11.72 a	<0.001	0.881

**Table 4:** Anesthesia of other marine and freshwater fish.

Specie	Habitat	Eugenol (mg/L)	MS-222 (mg/L)	Source
*Silurus asotus* (far eastern catfish)	Freshwater	500	600	[35]
*Lateolabrax maculatus* (Chinese sea bass)	Marine	6	30	[36]
*Takifugu obscurus* (river pufferfish)	Freshwater	–	150–175	[37]
*Scophthalmus maximus* (turbot)	Marine		60	[38]
*Sparus aurata* (gilthead sea bream)	Marine	1	60	[39]
*Centropomus undecimalis* (common snook)	Marine	50	–	[40]
*Geophagus brasiliensis* (pearl cichlid)	Freshwater	217	294	[41]
*Poecilia reticulata* (guppy)	Freshwater	50–75	–	[42]
*Oreochromis niloticus* (Nile tilapia)	Freshwater	90	300	[43]
*Pseudoplatystoma corruscans* (spotted sorubim)	Freshwater	75	–	[44]
*Oplegnathus punctatus* (spotted knifejaw)	Marine	35	80	[45]

## Data Availability

The original contributions presented in this study are included in the article. Further inquiries can be directed to the corresponding author(s).
